# Development of an MRI Radiomic Machine-Learning Model to Predict Triple-Negative Breast Cancer Based on Fibroglandular Tissue of the Contralateral Unaffected Breast in Breast Cancer Patients

**DOI:** 10.3390/cancers16203480

**Published:** 2024-10-14

**Authors:** Roberto Lo Gullo, Rosa Elena Ochoa-Albiztegui, Jayasree Chakraborty, Sunitha B. Thakur, Mark Robson, Maxine S. Jochelson, Keitha Varela, Daphne Resch, Sarah Eskreis-Winkler, Katja Pinker

**Affiliations:** 1 Department of Radiology, Columbia University Irving Medical Center, Vagelos College of Physicians and Surgeons, New York, NY 10065, USA; 2 Department of Radiology, Memorial Sloan Kettering Cancer Center, New York, NY 10065, USAthakurs@mskcc.org (S.B.T.);; 3 Department of Surgery, Memorial Sloan Kettering Cancer Center, New York, NY 10065, USA; 4 Department of Medical Physics, Memorial Sloan Kettering Cancer Center, New York, NY 10065, USA; 5 Department of Medicine, Memorial Sloan Kettering Cancer Center, New York, NY 10065, USA; 6 CUNY School of Medicine, New York, NY 10031, USA; 7 Medical School, Sigmund Freud University, A-1020 Vienna, Austria

**Keywords:** breast cancer, triple-negative breast cancer, radiomics, fibroglandular tissue

## Abstract

**Simple Summary:**

Triple-negative breast cancer is the most aggressive breast cancer subtype. However, women at risk for developing triple-negative breast cancer may not be identified by existing risk models. Thus, we present a study to determine if triple-negative breast cancer can be predicted based on a radiomic analysis and the machine-learning features of the fibroglandular tissue of the contralateral unaffected breast. Our initial results indicate that this approach can be used to predict triple-negative breast cancer. In the future, triple-negative breast-cancer-specific models may be implemented in the screening workflow to identify those women who are at elevated risk for triple-negative breast cancer specifically, for whom early detection and treatment are most essential.

**Abstract:**

Aim: The purpose of this study was to develop a radiomic-based machine-learning model to predict triple-negative breast cancer (TNBC) based on the contralateral unaffected breast’s fibroglandular tissue (FGT) in breast cancer patients. Materials and methods: This study retrospectively included 541 patients (mean age, 51 years; range, 26–82) who underwent a screening breast MRI between November 2016 and September 2018 and who were subsequently diagnosed with biopsy-confirmed, treatment-naïve breast cancer. Patients were divided into training (*n* = 250) and validation (*n* = 291) sets. In the training set, 132 radiomic features were extracted using the open-source CERR platform. Following feature selection, the final prediction model was created, based on a support vector machine with a polynomial kernel of order 2. Results: In the validation set, the final prediction model, which included four radiomic features, achieved an F1 score of 0.66, an area under the curve of 0.71, a sensitivity of 54% [47–60%], a specificity of 74% [65–84%], a positive predictive value of 84% [78–90%], and a negative predictive value of 39% [31–47%]. Conclusions: TNBC can be predicted based on radiomic features extracted from the FGT of the contralateral unaffected breast of patients, suggesting the potential for risk prediction specific to TNBC.

## 1. Introduction

Accurate risk stratification is pivotal for effective breast cancer prevention. Presently, women whose cumulative lifetime risk exceeds 20% are offered intensified high-risk screening with breast magnetic resonance imaging (MRI) in addition to yearly mammography [1]. Additionally, pharmacological risk reduction using tamoxifen or raloxifene hormonal therapy is offered to women whose five-year predicted breast cancer incidence exceeds 1.67% and women who have specific diagnoses, such as lobular carcinoma in situ, atypical hyperplasia, or ductal carcinoma in situ.

Currently, several models are available to predict breast cancer risk. The American Cancer Society recommends models that incorporate first- and second-degree family history in particular (i.e., Claus, Tyrer–Cuzick, and BRCAPRO). However, the consistent and reliable calculation of individual breast cancer risk with these models remains challenging [2] and significant variability exists in determining who would have a cumulative lifetime risk exceeding 20% [3]. Moreover, it must be considered that breast cancer is not a single entity but an amalgam of several different cancer subtypes, but to date, models are only focused on simply predicting breast cancer as a single entity and not the specific subtype. Breast cancers can be defined as hormone receptor-positive (HR-positive), human epidermal growth factor receptor 2 (HER2)-driven, or triple-negative (neither HR-positive nor HER2-driven). Of these subtypes, triple-negative breast cancer (TNBC) is associated with a shorter survival time [4]. However, given that the HR-positive disease is the most prevalent subtype by far, most epidemiological risk factors, as well as the polygenic risk score based on large genome-wide association studies, are conditioned to predict HR-positive, leading to the possible under-prediction of other subtypes. Known TNBC risk factors include germline mutation in the *BRCA1* gene; rare germline mutations in the *PALB2*, *RAD51C*, and *RAD51D* genes; and African American and West African descent [5,6,7,8]. Additionally, as some specific single nucleotide polymorphisms are associated with increased risk of HR-negative cancers, an HR-negative polygenic risk score is likely associated with TNBC. High mammographic breast density and background parenchymal enhancement on breast MRI also appear to be linked to both HR-positive cancer and TNBC [9,10,11]. The associations between cancer risk, mammographic density, and background parenchymal enhancement confirm the usefulness of imaging biomarkers for risk assessment, even if the biological mechanisms by which these characteristics lead to risk are poorly defined.

Recently, in addition to advanced imaging techniques, quantitative image analysis, incorporating a large number of features and integrating artificial intelligence or machine learning—an approach commonly known as radiomics—has created new research avenues [12]. Fundamentally, radiomics extracts features, representing intensity, shape, size, and/or texture, reflecting underlying pathophysiology. The central premise of radiomics is that biomedical images are the product of processes occurring at genetic and molecular levels and can be mined to identify features associated with specific cancers as well as cancer subtypes. In the case of TNBC, mined image features may be combined with known TNBC risk factors, improving the identification of women at risk for this most aggressive breast cancer subtype. Recent studies have demonstrated the growing application of radiomics in predicting TNBC. For instance, Leithner et al. utilized radiomic features extracted from dynamic contrast-enhanced MRI (DCE-MRI) and apparent diffusion coefficient (ADC) maps, achieving an area under the curve (AUC) of 0.86 for distinguishing TNBC from other subtypes [13]. Additionally, Zhang et al. compared different machine-learning models, finding that convolutional neural networks (CNNs) achieved up to 91% accuracy in classifying TNBC, outperforming convolutional long short-term memory models [14]. Yin et al. further advanced the field by using preoperative multiparametric MRI with a modified ResNet18 architecture, where CNN models based on post-contrast T1-weighted images achieved the highest AUCs (0.76−0.92) compared to CNN models based on ADC maps and T2-weighted images [15]. These studies underscore the effectiveness of radiomics in enhancing TNBC prediction and highlight the need for continued research in this area.

The purpose of this study was to develop a radiomic-based machine-learning model to predict TNBC status based on the contralateral unaffected breast’s fibroglandular tissue (FGT) in breast cancer patients. This is the first necessary step towards the long-term goal of defining a subtype-specific risk score, incorporating not only imaging features but also clinical and genetic data to identify women who are at risk for TNBC, the most aggressive breast cancer subtype, and thereby potentially reduce the number of TNBC-related deaths.

## 2. Materials and Methods

### 2.1. Patients

This was an institutional review board-approved and Health Insurance Portability and Accountability Act-compliant retrospective study, for which the need for written informed consent was waived. A total of 600 patients who underwent breast MRI between November 2016 and September 2018, doing so as part of our institution’s established screening program for patients at increased risk of breast cancer, and who were then diagnosed with treatment-naïve breast cancer (TNBC or non-TNBC), were identified. After excluding patients with bilateral disease (previous or concurrent) and patients with previous contralateral surgery (e.g., mastectomy, lumpectomy, mastopexy, or reduction mammoplasty), the final study sample comprised 541 patients with treatment-naïve breast cancer who were randomly divided into a training set of 250 patients and a validation set of 291 patients. Out of 250 in the training set, 156 patients (62%) had TNBC, and out of 209 patients in the validation set, 209 patients (72%) had TNBC.

### 2.2. Histopathologic Analysis

HR expression (via the detection of estrogen and progesteron receptors [ER, PR]) and HER2 expression were evaluated using surgical specimens or percutaneous biopsy (if the patient underwent neoadjuvant chemotherapy) according to the 2018 American Society of Clinical Oncology/College of American Pathologists guidelines [16,17]. ER/PR expression > 1% based on immunohistochemistry was considered positive. HER2 status was considered negative if the staining score was 0 or 1+, equivocal if the staining score was 2+, and positive if the staining score was 3+. Tumors with equivocal HER2 status were evaluated using fluorescence in situ hybridization (FISH) and considered positive if HER2 gene amplification was observed and negative if no gene amplification was observed. In the case of any outside biopsies, all pathologic results from outside biopsies were reviewed at our institution.

### 2.3. Imaging Acquisition

Breast MRI was performed using a 1.5 Tesla (GE Healthcare MR450; GE Healthcare, Chicago, IL, USA) or 3 Tesla (GE Healthcare MR750) scanner using an 8- or 16- channel phase-arrayed breast coil. All patients underwent a state-of-the-art breast MRI protocol comprising the following sequences: (a) Axial T2-weighted turbo spin-echo imaging with fat suppression (repetition time/echo time [TR/TE], 4091/102 ms; field of view [FOV], 340 mm; number of slices, 34; slice thickness, 3 mm; flip angle 111°; matrix, 288 × 224; number of excitations [NEX], 2; and acquisition time, 4 min 15 s). (b) Diffusion-weighted imaging (DWI) using three-acquisition trace diffusion-weighted single-shot echo-planar imaging with inversion recovery fat suppression (TR/TE/inversion time [TI], 8000/59/210 ms; dual shim volumes placed over both breasts to optimize poor magnetic field [B0] homogeneity; FOV, 360 × 196 mm; number of slices, 24–28; slice thickness, 5 mm; matrix, 192 × 192; b-values, 0 and 800 s/mm^2^; NEX, 3; and acquisition time, 3 min 36 s). (c) DCE-MRI using an axial 3D T1-weighted volume image breast assessment (VIBRANT) gradient echo imaging both before and after (three time points spaced 60 s apart) a contrast administration injection of 0.1 mmol/kg of gadopentetate dimeglumine (Magnevist; Bayer Corporation, Whippany, NJ, USA) (TR/TE, 4.3/2.1 ms; flip angle, 10°; NEX, 1; matrix, 320 × 320; FOV, 280−300 mm; number of slices, 200−300; slice thickness, 1 mm; and acquisition time, 2 min 15 s). Parallel imaging using the array spatial sensitivity encoding technique (ASSET) was applied during DWI and DCE-MRI.

### 2.4. Image Segmentation

**Figure 1:**
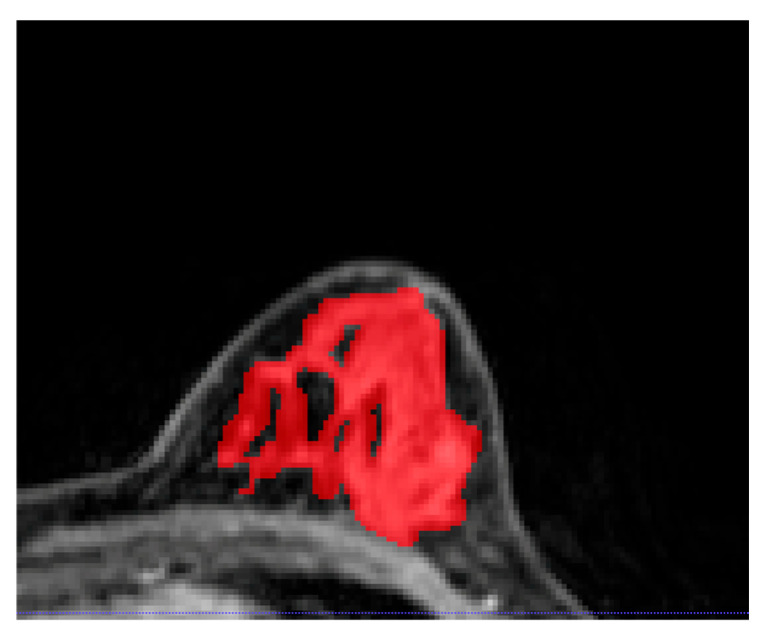
An example of fibroglandular tissue segmentation from the contralateral healthy breast on post-contrast T1-weighted imaging. Interspersed as well as surrounding subcutaneous adipose tissue were excluded from the segmentation.

Images from the DCE-MRI T1-weighted fat-saturated first post-contrast sequence were loaded as Digital Imaging and Communications in Medicine (DICOM) files into OsiriX (Pixmeo, Switzerland), an open-source image processing tool [18]. The semi-automated 2D segmentation of FGT of the contralateral unaffected breast was performed as follows: first, thresholding or clustering was performed using ITK-SNAP software (version, 3.6.0) (GNU General Public License, 2004) [19] to include the entire FGT (excluding fatty tissue); subsequently, manual corrections were made by two dedicated breast radiologists (E.O-A. and R.L.G., with 2 and 9 years of experience, respectively) (Figure 1).

### 2.5. Radiomics Analysis, Machine Learning, and Prediction Model Building

To avoid overfitting, the study dataset was separated into training and validation sets; the training set was used for feature selection and model development, while the validation set was used for assessing model performance.

Regarding feature selection and model development, following FGT segmentation, a total of 132 radiomic features were extracted via texture analysis as potential correlates of TNBC using the open-source Computational Environment for Radiological Research (CERR) platform [20]. The extracted radiomic features included 22 first-order features, 26 features based on the gray-level cooccurrence matrix (GLCM), 16 features based on the run length matrix (RLM), 16 features based on the size zone matrix (SZM), 17 features based on the neighborhood gray-level dependence matrix (NGLDM), 5 features based on the neighborhood gray tone difference matrix (NGTDM), and 30 features based on 3D Minkowski functionals. The intensity value was quantized into 32 intensity values and one interpixel distance was considered to compute second- and higher-order features. A radiomic model was then designed, using a support vector machine, to predict the risk of TNBC in the training set. A univariate analysis with the Wilcoxon-rank-sum test was performed to determine which radiomic features are significantly associated with TNBC. Of the remaining features, a univariate analysis was performed to reduce the set of features to those with an area under the receiver operating characteristic (ROC) curve (AUC) of >0.6. Minimum redundancy maximum relevance-based feature selection, followed by forward selection with 10-fold cross-validation, was then applied to select the final features. The final prediction model was designed using a support vector machine with a polynomial kernel of order 2, a gamma value of 0.0043, and a box constraint of 1. The parameters of the classifier were selected by optimizing the model with the training data; the parameters that obtained the best AUC with 10-fold cross-validation were chosen. We explored linear, radial basis function, and polynomial kernels. Similarly, the gamma value was explored within a range of values from 2^−10^ to 2^3^. Since the data were slightly imbalanced, to handle this issue, we applied cost-sensitive learning. Accordingly, while designing the model, instead of using equal cost to penalize wrong classification for both rare and abundant classes, a higher cost was assigned for the non-TNBC group (the rare class) compared to the TNBC group (the abundant class). The output of this classifier provided a TNBC risk score (TNBC-RS) from 0 to 1, corresponding to the level of confidence that a patient has TNBC.

Regarding the assessment of the performance of the model, F1 score, AUC, sensitivity, specificity, positive predictive value (PPV), and negative predictive value (NPV) values with corresponding 95% confidence intervals (CIs) were determined in the validation set.

## 3. Results

### 3.1. Clinical–Pathologic Characteristics

All patients were female regarding gender identity and sex assigned at birth. The mean age at diagnosis was 51 years (range, 26–82). Of the 250 patients in the training set (mean age, 50 years; range, 26–82), 156 were TNBC patients and 94 were non-TNBC patients. Of the 291 patients in the validation set (mean age, 51 years; range, 26–82), 209 were TNBC patients and 82 were non-TNBC patients.

### 3.2. Significant Radiomic Features

Of the 132 radiomic features that were extracted, 65 features were significantly different between TNBC and non-TNBC patients based on an AUC cut-off of >0.6. The significant features included 7 first-order features, 19 features based on the GLCM, 8 features based on the RLM, 8 features based on the SZM, 10 features based on the NGLDM, 3 features based on the NGTDM, and 10 features based on 3D Minkowski functionals.

### 3.3. Model Performance

**Figure 2:**
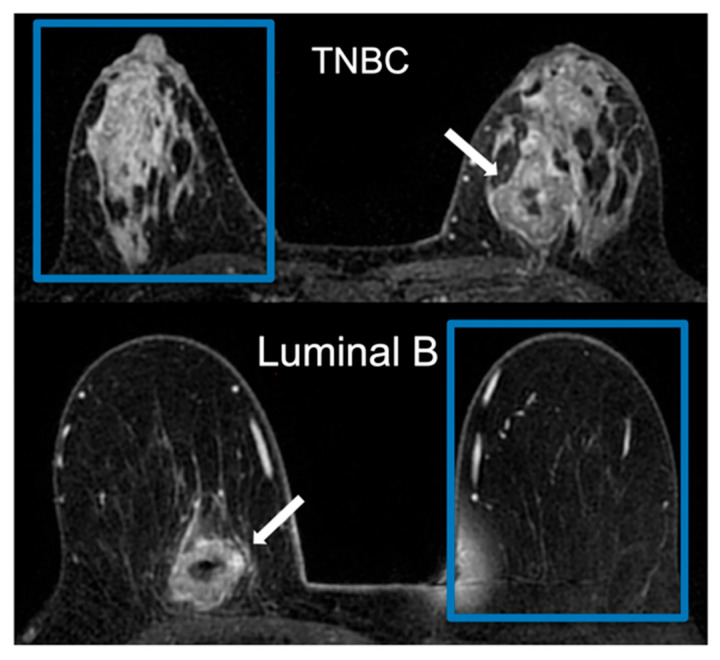
Example of a post-contrast axial T1-weighted image depicting an irregular lesion in the mid-inner quadrant of the left breast, which was correctly predicted by our radiomic machine-learning model as triple-negative breast cancer (TNBC), and an example of a post-contrast axial T1-weighted image depicting an irregular lesion in the lower-inner quadrant of the right, which was correctly predicted by our radiomic machine-learning model as luminal B cancer (ER-positive, PR-negative, and HER2-positive breast cancer). The fibroglandular tissue of the contralateral breast was segmented as in Figure 1.

**Figure 3:**
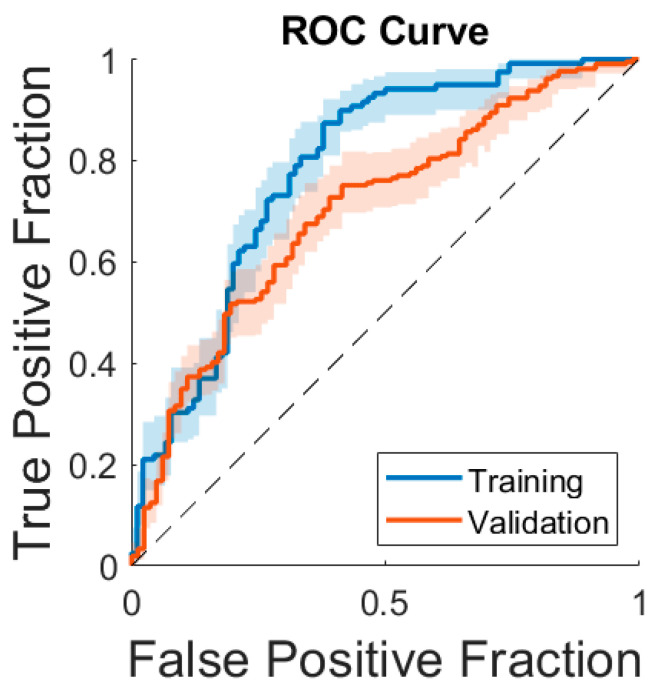
A comparison of the receiver operating characteristic (ROC) curves of the radiomic machine-learning model to predict the development of triple-negative breast cancer in the training set vs. the validation set. The true-positive rate (sensitivity) on the y-axis is plotted against the false-positive rate (100-specificity) on the x-axis.

After feature selection, four features were used for the model: min intensity, RLM-based long-run high-gray-level emphasis, SZM-based size zone non-uniformity normalized, and NGLDM-based high-dependence low gray emphasis. The model achieved an F1 score of 0.66, an AUC of 0.71, a sensitivity of 54% [47–60%], a specificity of 74% [65–84%], a PPV of 84% [78–90%], and an NPV of 39% [31–47%] in the validation set (Table 1). Figure 2 illustrates two cases of similar-appearing breast cancers (TNBC and luminal B) by MRI that were correctly predicted by the model. Figure 3 compares the model’s ROC curve in the validation set to its ROC curve in the training set.

## 4. Discussion

We present a study to determine if TNBC can be predicted based on radiomic analysis and machine-learning features of FGT of the contralateral unaffected breast. Indeed, our results show that our radiomic machine-learning model based on FGT of the contralateral unaffected breast can predict TNBC, with an AUC of 0.71. We believe that this study holds significance because, until now, risk models have been inconsistent in their performance and primarily concentrate on factors linked to HR-positive breast cancer instead of those linked to other less common breast cancer subtypes (HER2-positive cancer and TNBC). This narrow focus is regrettable, considering that TNBC is the most aggressive breast cancer subtype but women at risk for developing TNBC may not be identified by existing risk models. We hope that the data shown herein can improve upon this.

TNBC is characterized by the absence of estrogen receptor, progesterone receptor, and HER2 expression (hence the name “triple negative”) and accounts for 15%–20% of breast cancer cases [5]. Compared to other breast cancer subtypes, non-metastatic TNBC is associated with shorter survival. Even with the administration of chemotherapy, the 5-year event-free or disease-free survival rate is 70%, and the 5-year overall survival rate is only 77% [21,22,23].

There are many known risk factors for breast cancer. TNBC in particular is most commonly found in premenopausal women under 40 years of age with a germline mutation in the *BRCA1* gene or in the *PALB2*, *RAD51C*, and *RAD51D* genes [5,6]. While well-known reproductive risk factors such as early menarche, nulliparity, and late age at first childbirth are linked with ER-positive disease, they are not linked with TNBC [4]. TNBC is also more common in women of African American or West African descent [7,8], who tend to receive diagnoses at more advanced disease stages, resulting in a 41% higher breast cancer mortality rate compared to white women, equating to a substantial difference of 8.8 deaths per 100,000 women [24,25]. These longstanding racial disparities along with the associated socioeconomic obstacles have been evident in breast cancer outcomes for over four decades [26]. Given that epidemiological risk factors for breast cancer seem to vary by subtype, it may be possible to develop a more targeted risk prediction model specifically tailored to predict the risk of TNBC. Implementing a more targeted high-risk classification predictor could also enhance access to early screening and supplementary imaging, helping to reduce mortality and address racial disparities.

In our study, we used both radiomics and machine learning (a subset of artificial intelligence). Regarding radiomics, several studies have shown the potential of MRI-based radiomics to distinguish between breast molecular subtypes. Leithner et al. [27] reported promising results in distinguishing between TNBC and luminal B cancer using radiomic features of contrast-enhanced MRI, showing an accuracy of 83.9%. Meanwhile, Wang et al. [28] found that a Fisher discriminant radiomic model to distinguish between TNBC and non-TNBC patients achieved an accuracy of 83.4% in the validation set. A meta-analysis of six studies, including 1223 patients [29], further confirmed that MRI-based radiomics exhibited excellent ability to confirm or exclude TNBC (AUC 0.88). Several studies using either machine learning or deep learning have shown promising results using features extracted from the tumor for breast cancer characterization and molecular subtyping [14,15,30,31,32,33]. Demircioglu et al. [30] used a volume-of-interest approach to radiomics and the extracted radiomic features were analyzed with machine learning. Their model achieved an AUC of 0.73 for classifying TNBC. Wu et al. [31] used a decision-tree machine-learning model, achieving an accuracy of 70.31% for molecular subtype classification in the validation set. With larger datasets becoming available for analysis, the field has moved towards using deep- rather than machine learning. Yin et al. [15] reported that CNN models based on post-contrast T1-weighted images, T2-weighted images, and ADC maps, respectively, achieved AUCs of 0.64–0.92 for molecular subtype classification, with better performance for separating TNBC or HER2-positive subtypes from other subtypes than for separating luminal A or luminal B from other subtypes. Zhang et al. [14] compared a conventional CNN with a convolutional long short-term memory network to distinguish between HR+/HER2−, HER2+, and TNBC subtypes, achieving accuracies of up to 91% using a transfer learning approach. The main difference between the studies mentioned and our current study is that the radiomic features in our research are extracted from healthy fibroglandular tissue of the contralateral breast, rather than from the tumor itself. This choice is based on our goal to use information from healthy breast tissue to predict the occurrence of triple-negative breast cancer, which could serve as a risk prediction tool for the future. Artificial intelligence-based risk prediction models have also been developed, showing the ability to enhance breast cancer risk prediction, but these have not focused on predicting the risk for specific breast cancer subtypes [34,35].

While the abovementioned radiomics and artificial intelligence studies focused on using imaging features from the tumor, it has also been shown in the literature that normal tissue characteristics identified by MRI can be used for distinguishing between molecular subtypes as well as for performing risk stratification. To distinguish TNBC from other molecular subtypes, Wang et al. [32] used features extracted from both background parenchymal enhancement and the tumor. The predictive models to distinguish TNBCs from other subtypes were based on support vector machines. While tumor-related features demonstrated an AUC of 0.78, the incorporation of background parenchymal enhancement-related features significantly increased the AUC to 0.88 (*p* < 0.01), with background parenchymal enhancement-related features being the most discriminating features. To predict breast cancer risk, King et al. [36] reported a significant increase in breast cancer risk among women with moderate-to-marked enhancement compared to minimal or mild enhancement, and the difference remained significant after correction for FGT. Niell et al. [37] quantitatively measured background parenchymal enhancement using a semi-automated segmentation algorithm for predicting subsequent breast cancer development. The percentage of breast tissue that enhanced above the 30% or 40% threshold relative to the total breast volume (background parenchymal enhancement %) on the first post-contrast sequence showed the highest AUCs of 0.84–0.85 for breast cancer prediction. Saha et al. [38] developed a machine-learning model using features extracted from background parenchymal enhancement of high-risk screening MRIs for predicting future breast cancer. The model was able to discriminate between patients who would develop a breast cancer from those who would not over a follow-up period of 2 years, with an AUC of 0.70, confirming that background parenchymal enhancement may potentially be used to further stratify risk in patients undergoing high-risk screening MRI. Elsewhere, Wang et al. [39] segmented the FGT from baseline MRI scans and the extracted features were used to assess association with breast cancer occurrence. This study included 4,553 women with extremely dense breast, and 122 who were diagnosed with breast cancer. A high volume of enhancing parenchyma on baseline DCE-MRI was associated with an increased occurrence of breast cancer (hazard ratio, 1.09; 95% CI: 1.01–1.18; *p* = 0.02).

To date, associations between normal tissue characteristics and the risk for a specific breast cancer subtype have not been reported in the literature. Our study provides initial results, showing that there is potential to predict the TNBC subtype from standard-of-care breast MRI by segmenting the FGT from the contralateral healthy breast. Our model achieved an F1 score of 0.66, an AUC of 0.71, a sensitivity of 54%, a specificity of 74%, a PPV of 84%, and an NPV of 39% for predicting TNBC in the validation cohort. Given that we are analyzing healthy tissue instead of tumor-specific features, it is understandable that our performance metrics are not as high as those reported in studies that focus on tumor-specific radiomic features. These initial results pave the way for building more robust risk prediction models by further incorporating clinical and genetic data linked to the development of TNBC such as ethnicity and *BRCA1*, *PALB2*, *RAD51C*, and *RAD51D* gene mutation status.

This study comes with several limitations. One limitation is the small sample size, which may impact the generalizability of the findings. While we were able to confirm our results in a validation set, a larger sample could provide more robust and reliable data. Additionally, instead of using data from healthy individuals who later developed breast cancer over time, we used data from patients currently diagnosed with breast cancer, which influenced our choice to examine the healthy contralateral breast. Notably, TNBC is more often diagnosed in younger women presenting with symptoms and for whom prior screening is often not available; thus, using data from healthy individuals who later develop breast cancer over time would have further reduced our sample size. A follow-up study that includes data from individuals without a cancer diagnosis and adequate follow-up could further validate our approach and confirm that an MRI of healthy tissue can be used to assess breast cancer risk effectively. Another limitation is that we did not integrate clinical, genetic, and epidemiological data into our radiomic machine-learning model; however, we anticipate addressing this in a future study involving a larger patient sample.

## 5. Conclusions

In conclusion, we present initial results indicating that a radiomic machine-learning model based on FGT of the contralateral unaffected breast can predict TNBC. In the future, TNBC-specific models may be implemented in the screening workflow to identify women who are at elevated risk for TNBC specifically, which carries the worst prognosis, for whom early detection and treatment are most essential.

## Figures and Tables

**Table 1:** Results from the final machine-learning model consisting of four features (min intensity, RLM-based long-run high-gray-level emphasis, SZM-based size zone non-uniformity normalized, and NGLDM-based high-dependence low gray emphasis) across training and validation sets.

	F1 Score	AUC	Sensitivity	Specificity	PPV	NPV
Training set (*n* = 250)	78% [71–86%]	78% [72–84%]	81% [74–88%]	67% [57–76%]	76% [69–84%]	72% [63–82%]
Validation set (*n* = 291)	66% [59–72%]	71% [63–76%]	54% [47–60%]	74% [65–84%]	84% [78–90%]	39% [31–47%]

Abbreviations: AUC, area under the curve; NGLDM, neighborhood gray-level dependence matrix; NPV, negative predictive value; PPV, positive predictive value; RLM, run length matrix; SZM, size zone matrix.

## Data Availability

The datasets generated during and/or analyzed during the current study are available from the corresponding author on reasonable request.
